# MetCap: a bioinformatics probe design pipeline for large-scale targeted metagenomics

**DOI:** 10.1186/s12859-015-0501-8

**Published:** 2015-02-28

**Authors:** Sandeep K Kushwaha, Lokeshwaran Manoharan, Tejashwari Meerupati, Katarina Hedlund, Dag Ahrén

**Affiliations:** Department of Biology, Lund University, Ecology Building, 223 62 Lund, Sweden; Bioinformatics Infrastructure for Life Sciences (BILS), Department of Biology, Lund University, Ecology Building, 223 62 Lund, Sweden

**Keywords:** Bioinformatics, Environmental sequencing, Functional genes, Metagenome, Probe design pipeline, Targeted metagenomics, Sequence capture, MetCap

## Abstract

**Background:**

Massive sequencing of genes from different environments has evolved metagenomics as central to enhancing the understanding of the wide diversity of micro-organisms and their roles in driving ecological processes. Reduced cost and high throughput sequencing has made large-scale projects achievable to a wider group of researchers, though complete metagenome sequencing is still a daunting task in terms of sequencing as well as the downstream bioinformatics analyses. Alternative approaches such as targeted amplicon sequencing requires custom PCR primer generation, and is not scalable to thousands of genes or gene families.

**Results:**

In this study, we are presenting a web-based tool called MetCap that circumvents the limitations of amplicon sequencing of multiple genes by designing probes that are suitable for large-scale targeted metagenomics sequencing studies. MetCap provides a novel approach to target thousands of genes and genomic regions that could be used in targeted metagenomics studies. Automatic analysis of user-defined sequences is performed, and probes specifically designed for metagenome studies are generated. To illustrate the advantage of a targeted metagenome approach, we have generated more than 300,000 probes that match more than 400,000 publicly available sequences related to carbon degradation, and used these probes for target sequencing in a soil metagenome study. The results show high enrichment of target genes and a successful capturing of the majority of gene families. MetCap is freely available to users from: http://soilecology.biol.lu.se/metcap/.

**Conclusion:**

MetCap is facilitating probe-based target enrichment as an easy and efficient alternative tool compared to complex primer-based enrichment for large-scale investigations of metagenomes. Our results have shown efficient large-scale target enrichment through MetCap-designed probes for a soil metagenome. The web service is suitable for any targeted metagenomics project that aims to study several genes simultaneously. The novel bioinformatics approach taken by the web service will enable researchers in microbial ecology to tap into the vast diversity of microbial communities using targeted metagenomics as a cost-effective alternative to whole metagenome sequencing.

**Electronic supplementary material:**

The online version of this article (doi:10.1186/s12859-015-0501-8) contains supplementary material, which is available to authorized users.

## Background

Microbial functional diversity is an area of interest and development due to their wide diversity and functioning in ecological processes [1,2]. Current knowledge of the key organisms behind the biological processes is scarce for the understanding of environment and climatic changes, bioremediation, symbiosis, biofuel production, medicine and agriculture productivity [3,4]. The high functional diversity is due to an extremely high diversity of microorganisms in our environment and it has been estimated that 16,000 to 8.3 million bacterial species can be found in one gram of non-contaminated soil [5]. To understand the contribution of different species in soil communities, next generation sequencing (NGS) enables us to sequence a larger part of a metagenome than traditional sequencing like Sanger sequencing. Nowadays, NGS is widely used in metagenomics due to more possibilities to identifying novel sequence with high-throughput yield, high molecular precision among individual sequences and cost effective [6]. Whole metagenome sequencing can be important for functional and taxonomical assessment of unexplored metagenomes. However, the amount of sequencing required for adequate coverage of a whole metagenome for analyzing a subset of genes is cumbersome in research studies [7,8]. Furthermore, downstream bioinformatics analyses can have difficulties in segregating large amount of non-target sequences from the targeted ones. Targeted metagenomics thus provide an alternative when studying specific gene families in metagenome communities [9]. Among the available techniques, amplicon sequencing based on PCR primers for target enrichment is an alternative approach to enrich certain genes of interest before sequencing. However, in contrast to targeting highly conserved regions such as 16S RNA, primer design for amplification of more variable genes in a metagenome, such as enzymes, is an intractable approach as it suffers from other difficulties such as PCR inhibitor and chimeras, amplicon length, cycle-numbers, specific amplification conditions [10,11]. In this context, sequence capture technique can make it possible to target regions of interest, while minimizing the fraction of off-targets on a large-scale. The sequence capture technique picks up DNA fragments of interest from a metagenomic DNA fragment pool through a user-designed set of probes [12]. This method has been utilized successfully in the field of medicine to preferentially sequence a targeted region of a genome [13]. The identification of potential probes is the primary requirement of sequence capture technique. There are several tools available for the generation of functional gene arrays (FGA) that have been used for various studies. Probes can be designed for a genome or specific set of genes through OligoArray [14], OligoWiz2.0 [15], OligoPicker [16], and YODA [17]. Hierarchical-Probe Design [18], PhylArray [19], ProDesign [20], CommOligo [21], Metabolic Design [22] and HiSpOD [23] were used in various metagenomic environmental studies. However, currently there is no publicly available probe generation pipeline for targeting multiple regions in metagenomic analyses. We are suggesting here that designing of multiple probes suitable for the sequence capture technique will open the doors for large-scale targeted metagenomic functional studies.

In the present study, the targeted metagenomics approach was applied to a soil metagenome to illustrate both the efficiency of this novel technique and the development of the open access probe design tool (MetCap). MetCap aims at achieving high probe coverage over a large amount of sequence data for targeted metagenomic studies. This web resource enables automatic probe design for sequencing of a large number of genes and gene families in a targeted metagenome using the sequence capture technology.

## Implementation

### Probe designing scheme

**Figure 1:**
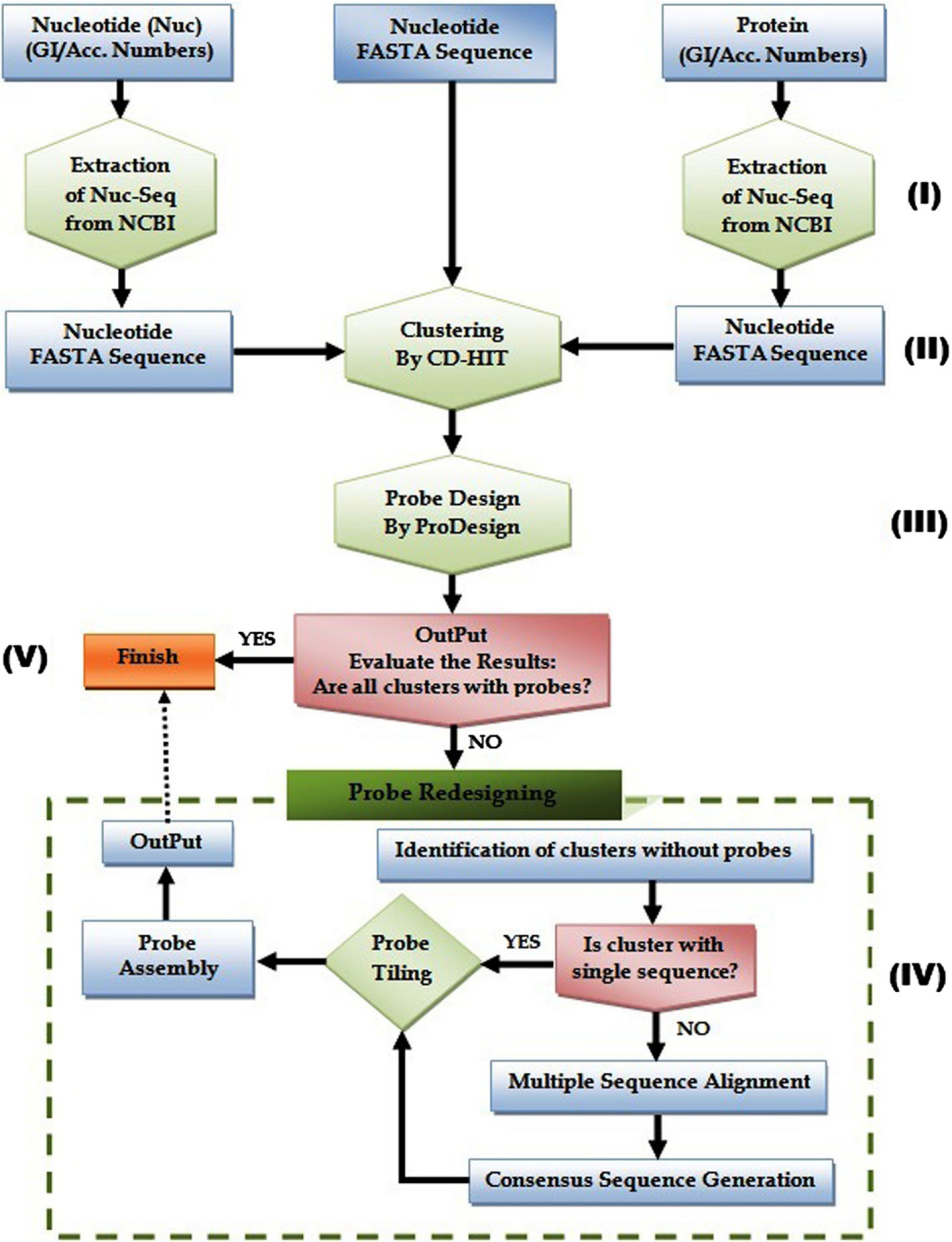
**Flowchart of the MetCap probe designing pipeline.**

Probe design is an important step in the sequence capture analysis, and the primary goal of the proposed tool is to design a set of probes for the majority of clusters from the input dataset. To achieve the objective, we test and used CD-HIT [24], ProDesign [20], Perl [25] and Bioperl [26] to develop MetCap. The probe design pipeline involved an extraction of a large amount of data from the National Center for Biotechnology Information (NCBI) based on user input, filtering of extracted data and intense processing for optimal probes with criteria adjusted specifically to fit metagenomic studies. The MetCap pipeline has five sequential steps of data processing (Figure 1).

#### Step-I nucleotide sequence extraction and submission

The first phase of the pipeline is involved in the extraction and submission of sequences. MetCap provides four different options for nucleotide sequence extraction from NCBI identifiers: The NCBI accession number (either as nucleotide id or as protein id) or GI number (nucleotide or protein). The nucleotide sequence extraction process uses Perl and Bioperl coupled with NCBI E-utilities [27]. MetCap is facilitating the single gene nucleotide sequence extraction from accession and GI numbers while long sequences that are likely to be genomic sequences such as contigs or scaffolds will be removed. MetCap provides an option to download the nucleotide sequences that was retrieved by the extraction process. MetCap provides two different options of sequence submission; firstly to execute the pipeline by the submission of the extracted nucleotide sequence through accession, or GI numbers from the extraction process as described above. Secondly to start the execution of the pipeline by direct submission of user-collected nucleotide sequences that can be processed directly in Step-II. This is the recommended approach whenever possible.

#### Step-II data clustering

The CD-HIT clustering software has been used for cluster identification on the basis of a given sequence identity threshold. CD-HIT clustering parameters (identity threshold, word-size, comparing stands (−r), global sequence identity (−G), best cluster identification (−g) and bandwidth of alignment (−b)) are interactive in the pipeline [24]. User can change these values in pipeline in order to explore the clustering. Default values for clustering in MetCap pipeline is set same as CD-HIT defaults values. This phase is computationally intense and critical for the probe design because each cluster will behave like an individual group, and each cluster can have hundreds of sequences.

#### Step-III probe design

The third phase designs the initial probes through ProDesign. ProDesign generates keywords from sequences then it matches the keywords with cluster sequences to find out the probe candidates for each cluster. ProDesign [20] was selected as the design tool since it is fast for a large number of sequences, easy to integrate into the pipeline, and highly efficient among sequences with moderate similarity between sequences that were used to design the probes.

#### Step-IV probe redesign

The fourth phase designs the probes for those clusters which were without probes after Step-III. MetCap can identify and separate the clusters for which the probe has not been designed by ProDesign, and will then check the number of sequences in each cluster. If a cluster has more than one sequence, a conventional probe design approach will be used. First, all the sequences of clusters will be aligned (multiple sequence alignment) and then a consensus sequence will be generated from the aligned sequences for each cluster at 90% sequence identity. Probe tiling will then be performed through a given length of probe over consensus sequences. Probe will be filtered through a given melting temperature and GC-content [28]. To increase the number of clusters with probes, direct sequence tiling and filtering on user-defined parameters was used for those clusters which have a single sequence because every cluster has its own relevance in targeted functional studies. All the generated probes will be added to the probes generated in Step-III. The MetCap pipeline also provides options to change the parameters for redesigning at step IV. Step-IV uses the same four parameters as in step-III; maximum expected number of probes per cluster, probe length, melting temperature and GC content. While, the same settings as in step-III are supposed to be used, there are some cases when probes are not generated at this step because there are no regions in the clusters that fulfill the criteria. Therefore, users can change the values of these parameters (increase/decrease) in step-IV to produce additional probes at the probe redesign step. Thus, the chance of designing probes for the clusters is significantly increased.

#### Step-V output and summary

MetCap generates output for each step, and the generated output was stored and summarized in a zip-compressed folder containing 6 files. Detailed descriptions of the file names and a summary of the file contents are given in Table 1.

**Table 1:** **List of MetCap generated files and their descriptions**

**S.N.**	**File Name**	**File Description**
1.	probe_run1_summary	Summary of initial probe generation
2.	probe_run2_summary	Summary of final probe generation
3.	probe_file1	Contains the initially (first) generated probes
4.	probe_file2	Contains all probes from both generations
5.	core_cluster.txt	Clustering file of submitted sequences
6.	probe_non_redundant.txt	Non-redundant set of generated probes

### Web implementation

The MetCap tool was installed on a Dell PowerEdge T320 Server E5-2430 with 6 Core processors of 2.20 GHz, and running on CentOS 6.5, 64 bits. The application is freely accessible from a web-interface which has been developed in PHP version 5.5.10 [29] and other freely available software such as CD-HIT [24] and ProDesign [20]. MetCap has two modes as web-interface. First is an interactive mode, where user can test and evaluate the results multiple times for small fraction of data from large dataset. Later, the user can submit all sequences together through non-interactive batch submission option and user notification via email. User can upload upto 100 MB file size through batch submission, which is large enough to upload all the sequences of particular gene family (approx. 50,000) like sequences of CAZy database. MetCap is using NCBI fasta format and other pseudo fasta format which is written in the help section of website. MetCap running time may be differ due to variable NCBI response time for sequence extraction. Running time of MetCap for probe generation may be significantly reduced through direct sequence submission.

### Sample and library preparation for sequence capture experiment

An agricultural soil sample was taken from a farm in Scania, South Sweden and DNA was extracted using a Nucleospin soil DNA isolation kit [30]. A DNA rapid library for sequence capture was prepared using Roche’s GS FLX rapid library preparation method. Probe-target hybridization was carried out through normal capture protocol according to the probe’s manufacturer (SeqCap EZ from NimbleGen) at 47°C and used with a slightly modified hybridization time of 24 hours instead of 72 hours (optimized for genomic regions with denser probes for targets) to reduce the stringency level of probes hybridizing to the DNA fragments [31]. Captured DNA reads were sequenced using the GS FLX Titanium system at the in-house 454 sequencing facility at the Department of Biology, Lund University.

### Similarity search and CAZy domain assignment

BLASTX similarity searches for sequenced reads were performed through mpiBLAST on resources provided by SNIC of UPPMAX [32]. BLASTX searches were used against a local targeted database, NCBI-NR and Uniprot databases [33]. A CAZymes Analysis Toolkit (CAT) was used for CAZy gene families assignment of the reads [34].

### Read assembly and mapping

GS *de novo* assembler was used for assembly of the sequenced reads whereas GS mapper was used for mapping of bacterial genomes and assembled reads [35]. BLAST Ring Image Generator (BRIG) was used for graphical representation of reads mapping to bacterial genomes [36].

## Results and discussion

MetCap is a web tool with open access that can generate thousands of targeted probes for large-scale metagenomic studies. Before designing MetCap, different available functional gene arrays generating software for environmental studies were compared and evaluated with parameters such as approaches used, types applied, capacity of the software, probe specificity checking criteria, platform and interface, current availability, and run-time for large datasets. In the evaluation, low probe coverage (i.e. number of clusters without probes) was the major problem in most of the large-scale probe designs. Short metagenomic sequences those with length shorter than 100–200 bases may be a reason for low probe coverage in some software, whereas extremely high and very low overlapping regions among the sequences of cluster could also be another reason for low probe coverage, because it might be difficult to extract the individual and group-specific seeds from sequences from these regions. The results of the evaluation are given in Additional file 1: Table S1. The comparison of FGA generating software showed that ProDesign [20] was the most efficient at generating probes that could match the target sequences as well as capture the functional diversity of highly similar genes from other organisms (i.e. paralogs and orthologs). ProDesign uses a spaced seed algorithm coupled with clustering, which is excellent for reducing the sequence data load and potential probe generation for a group of sequences. Probe specificity checking through input sequences makes it highly time efficient as well. Moreover, it generates group-specific probes with gaps less than 3 bases between seed words for the exploration of other sequences of a group [20]. Generated probes were found efficient for capturing of conserved and variable regions of a group, which was further verified through multiple sequence alignment. The downside of ProDesign is low probe coverage for clusters in metagenomic sequence dataset because ProDesign is not very efficient to generate probe when groups contain highly divergent sequences, or when there are highly similar sequences found in between groups [20]. As in many metagenome studies, the number of sequences for each target was very large and the diversity between the sequences was very high. Consequently, high probe coverage is a major challenge for large-scale metagenomics data sets.

MetCap uses a hybrid approach for large-scale data set processing and can be used for probe design with any set of user-defined sequences that are targeted in environmental metagenomics samples. In MetCap, initial probes are designed through ProDesign software. In order to increase the probe coverage for clusters, MetCap identifies the clusters without probes, and collects all the sequences of each cluster separately from user-defined sequences for probe regeneration (Step-IV, above). MetCap approach is producing higher number of cluster with probes (Additional file 2: Table S2). The probes generated through MetCap are not affected by differences in abundance, since MetCap uses a clustering and probe selection approach. Any highly abundant set of sequences will be clustered and treated as one sequence. Probe selection is focused on maximizing the coverage of a cluster to enhance the hybridization chances. All the generated clusters will produce the same number of probes with similar properties as defined by the user. The optimal number of probes is also an important factor for large-scale sequence capture, but it is very hard to speculate on the exact number of probes for the capturing of a whole cluster. MetCap can generate different numbers of probes for each cluster from different regions to facilitate efficient capturing. The MetCap pipeline generates the probes in two steps and provides a summary of each step. MetCap Generated output (Table 1) can be downloaded when the analysis is finished.

Clustering (Step-II) and probe designing (Step-III and Step-IV) can be setup different because each step of pipeline is independent from each other. Initially, MetCap is performing clustering over thousands of sequences for probe generation. So, clustering threshold is crucial step because it can affect the number of cluster with probes. Therefore, we recommend using pipeline on default settings and evaluate (number of clusters with probes, number of probe per cluster) the output for each change setting from default settings. So, user can optimize the probe designing parameters to achieve maximum clusters with probes. MetCap also calculates the number of NimbleGen probe synthetic cycles for synthesis of each of the generated probes for cost estimation [37]. It should be noted that MetCap is independent from the sequencing platform, and that the sequence capture protocols are available for other sequencing platforms [38]. We demonstrated the effectiveness of the MetCap approach through a large-scale probe generation for an important and relatively well studied biological process: carbohydrate decomposition. To do this, three major public databases CAZy [39], FOLy (now part of the CAZy database as class: Auxiliary activities) [40] and Merops [41] were selected, and a local database was created. This contained the four enzyme classes and one associated module of the CAZy database, 10 families of the FOLy database, and secretory sequences through the signalP tool [42] from nine classes of proteolytic enzymes of the Merops database (Additional file 3: Table S3). To facilitate the time consuming tasks of sequence collection in large-scale metagenomic studies, MetCap allows users to extract nucleotide sequence through accession number and GI number of nucleotide and protein and also provides the facility to download nucleotide sequences for user verification. In this study, 396,297 nucleotide sequences were extracted through the pipeline and used as a proof of concept. A list of group-wise collected sequences and generated probes from databases are given as Additional file 3: Table S3. In total, 316,617 probes were produced from these extracted nucleotide sequences in this study with the following criteria: length (50mer), GC contents (35–65), melting temperature (55–65), and 3 probes per cluster on 90% cluster similarity.

**Figure 2:**
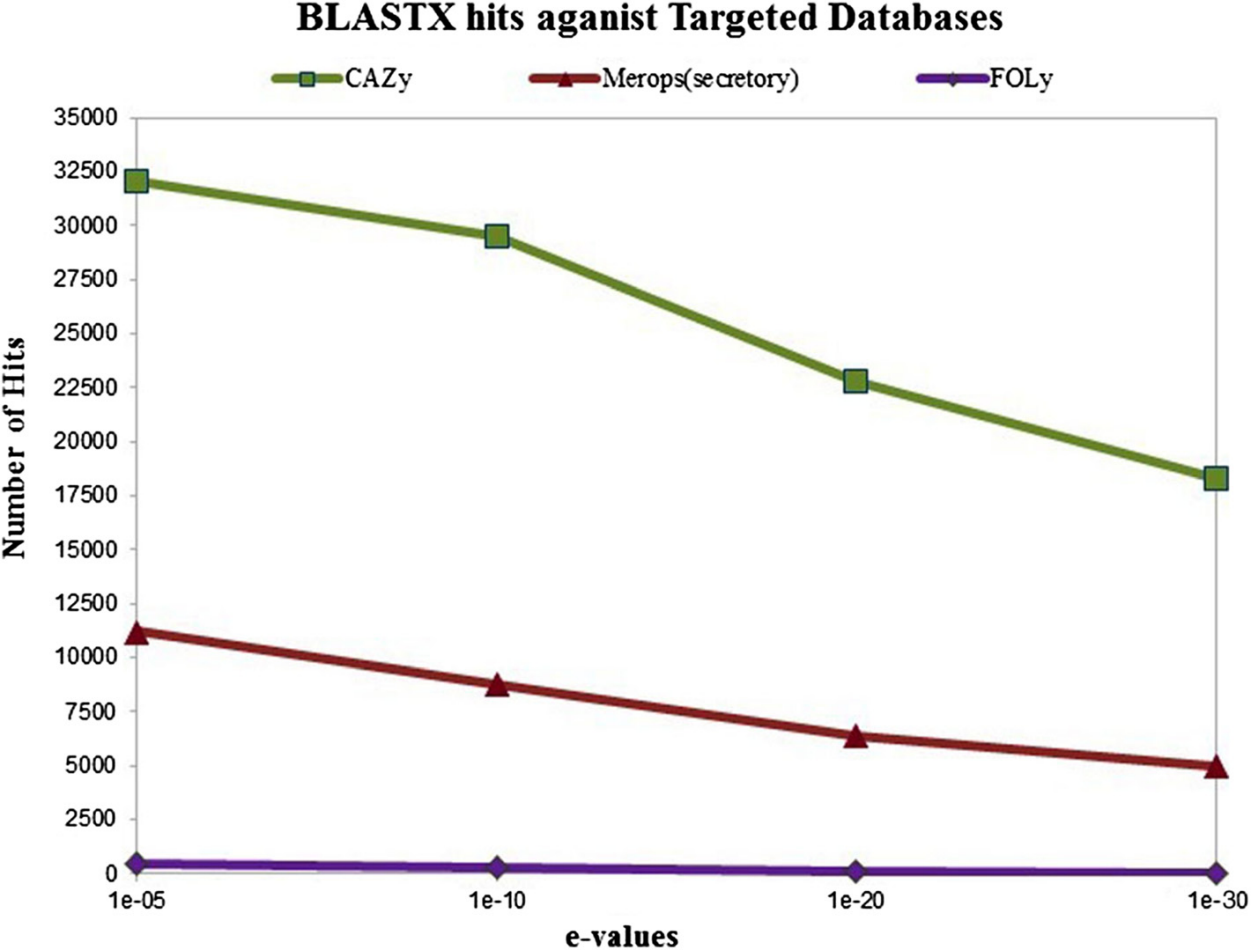
**BLASTX hits against targeted databases on different e-values.**

**Figure 3:**
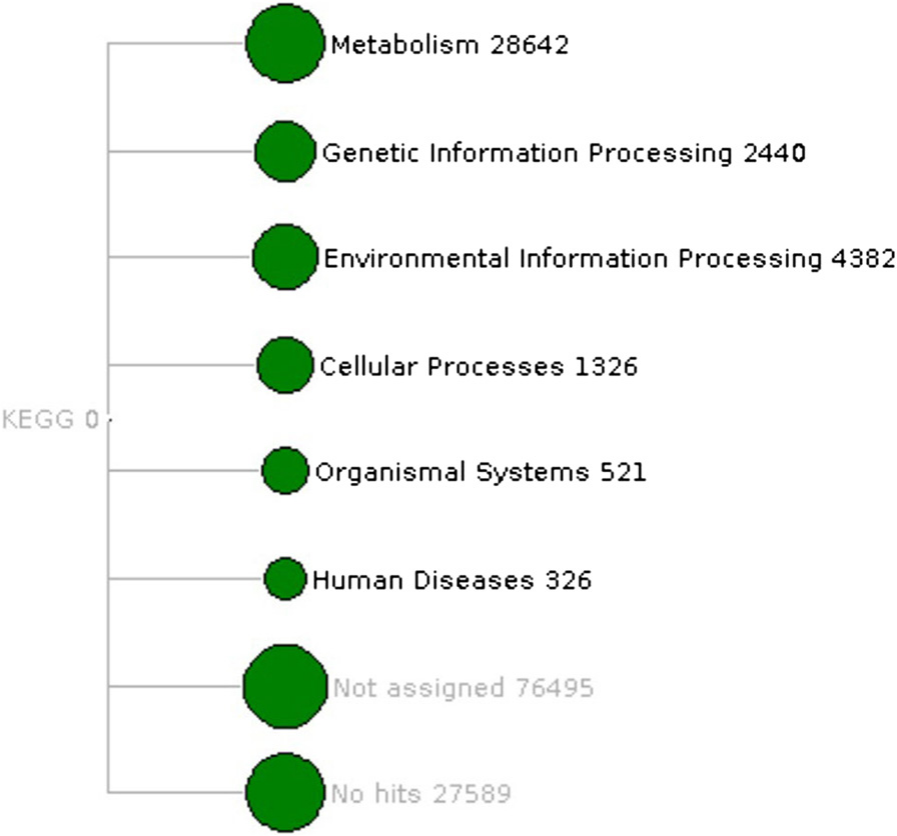
**Functional profile in sequenced reads through MEGAN.**

**Figure 4:**
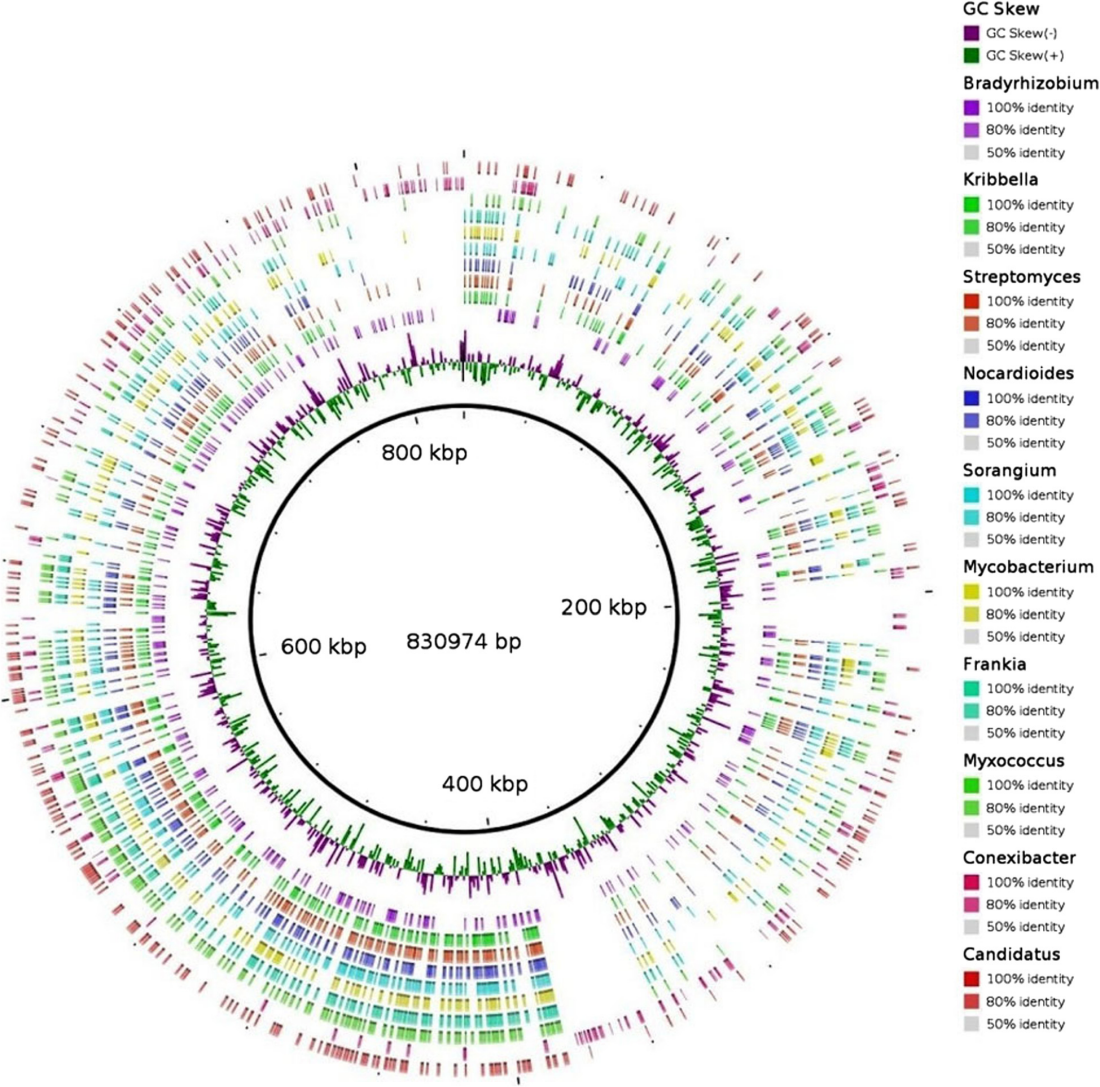
**Mapping of sequenced reads over 10 bacterial genomes and full description of abbreviations along with mapping percentage.** Bradyrhizobium (Bradyrhizobium japonicum USDA 6, 17.8%), Kribbella (Kribbella flavida DSM 17836, 13.9%), Streptomyces (Streptomyces coelicolor A3(2), 13.4%), Nocardioides (Nocardioides sp. JS614, 10.5%), Sorangium (Sorangium cellulosum So0157-2, 10.0%), Mycobacterium (Mycobacterium smegmatis JS623, 8.8%), Frankia (Frankia sp. EAN1, 7.7%), Myxococcus (Myxococcus xanthus DK 1622, 5.4%), Conexibacter (Conexibacter woesei DSM 14684, 5.3%), Candidatus (Candidatus Solibacter usitatus Ellin6076, 3.2%).

The next important step of the sequence-capture technique is the hybridization between designed probes and DNA fragments. DNA was extracted from agricultural soil and a DNA library was prepared for probe-target hybridization. 138,970 captured reads were sequenced from the 454 sequencing platform. 9,772 sequences failed in quality control. 129,189 reads were used in further analysis (Table 2). Sequenced reads are publicly available through metagenomic sequence repository MGRAST [43] with metagenome id 4529373.3. The read assignment was done through BLASTX searches against the different databases (Targeted databases, NCBI-NR and Uniprot) and capture efficiency was calculated (Table 3). The capture efficiency was defined as the fraction (%) of on target reads that matched to the target database sequences (proteins) which were used for designing the capture probes. The reads were decided as on-target when they had e-values lower than 1e-10. Metagenomic sequence capture efficiency was estimated 29.86% for the sample. The number of BLASTX hits against targeted databases differed between the databases (Figure 2) due to the different sizes of the targeted databases (Additional file 3: Table S3). The CAZy database has the maximum number of BLASTX hit, whereas the FOLy database has the least number of hits. BLAST searches have shown that a large number of sequences were captured during the experimental hybridization that were not found in our targeted databases or in public databases NCBI-NR and Uniprot. The MEGAN software [44] analysis has yielded very similar results compared to BLASTX for functional assignment of reads, which is verifying the capturing of sequences with unknown identities. 27,589 no-hits and 76,495 not-assigned reads (Figure 3) were found in the MEGAN metabolic analysis based on the NCBI-NR BLASTX result. The captured reads belong to hundreds of microbial genomes with highly variable abundance. The genomic distribution of captured sequenced reads can be analyzed among different abundant microbial species. Mapping of reads against the bacterial genomes showed that the distribution of reads over individual genome as well as bacterial genome were similar to each other for captured reads. A list of the most abundant bacterial species was generated on the basis of read numbers through the taxonomic analysis result of the MG-RAST pipeline [43], and the most abundant species from a wide range of phyla were selected. The GS *de novo* assembler [35] of 454 sequencing platform was used for assembling the sequenced soil metagenome. The assembled reads were mapped to the 10 most abundant bacterial genomes found in this experiment. Maximum mapping (17%) was achieved for *Bradyrhizobium japonicum USDA-6* over the assembled reads. The assembled reads mapped over bacterial genomes were shown in Figure 4 through BRIG software [36].

**Table 2:** **Quality control results**

Number of sequenced reads	138,970
Number of sequenced reads after filtering	129,198
Number of reads failed in quality control	9,772
Base count among filtered reads (bps)	44,281,009
Mean sequence length (bps)	342 ± 212
Mean GC percent	63 ± 5%

**Table 3:** **Comparative table of BLAST hits and capture efficiency against different databases on the e-value 1e-10**

**S.N.**	**Databases**	**BLAST**	**BLAST Hits**	**Read Matching (%)**
1.	Targeted Nucleotide Database	BLASTN	27,131	20.99
2.	Targeted Protein Database	BLASTX	37,329	28.89
3.	NCBI-NR	BLASTX	66,822	51.72
4.	Uniprot	BLASTX	65,903	51.00

**Figure 5:**
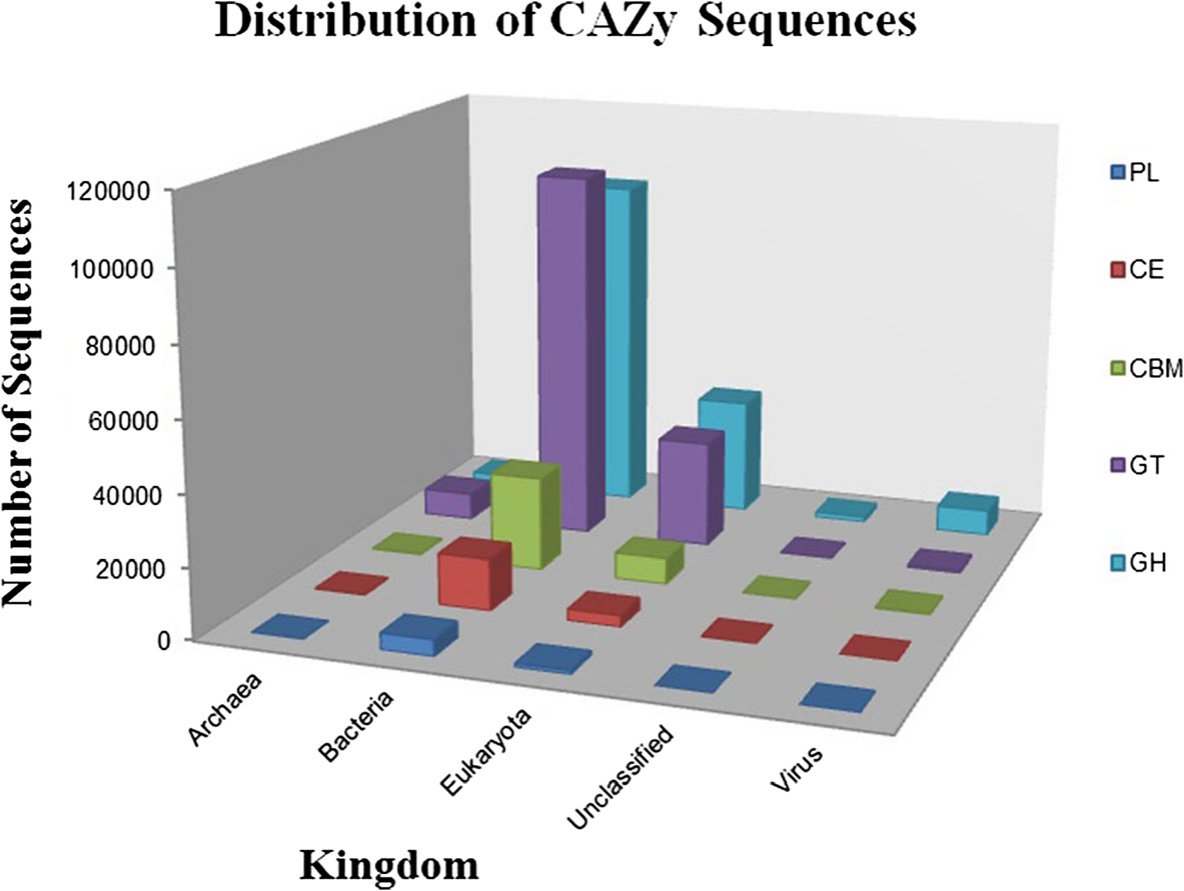
**Distribution of kingdom for CAZy families in collected sequences.**

**Figure 6:**
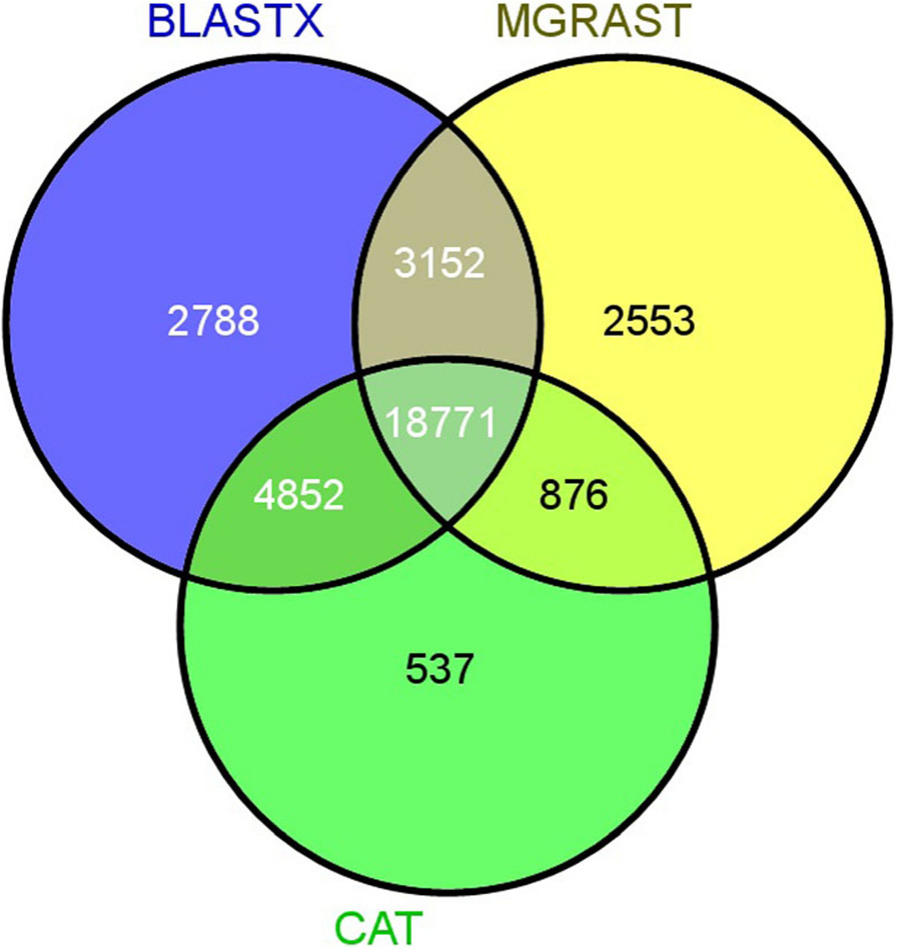
**Venn diagram of reads assigned by three methods.**

**Figure 7:**
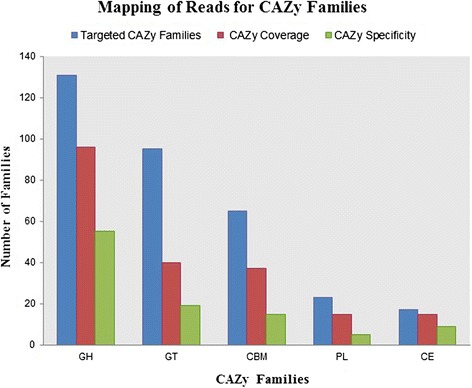
**Mapping of reads for CAZy families through read assignment.**

In this study, carbohydrate decomposing genes from microorganisms in soil were targeted as a demonstration of the potential of designed probes with the MetCap tool. Among the downloaded sequences (Additional file 3: Table S3), 348,316 sequences belong to the CAZy database from four major families: Glycoside Hydrolases (142,724), Glycosyl Transferases (146,087), Carbohydrate Esterases (18,286), Polysaccharide Lyases (5,859), and an associated module, Carbohydrate binding-modules (35,360) and 258,544 probes have been designed from these sequences. The distribution of sequences of targeted CAZy families with respect to kingdom was shown in Figure 5, though only targeted and highly similar sequences related to targets were considered as on-target in this study. To investigate the effect of sequence similarity cut-off for identification of on-targets and off-targets on a large-scale, we used three independent methods for assigning on-targets. The three methods were CAZy similarity search through BLASTX against the targeted database, CAZy Domain matching [34], and CAZy EC number matching [43] for each read. In the read assignment, reads that hit with e-values less than 1e-10 were considered for BLASTX. Domain identification in reads was done through CAZymes Analysis Toolkit (CAT) when the e-value was less than 1e-10. CAT tool uses BLAST similarity search to identify the best hits for query sequence in the CAZy database and then finds a link between CAZy families and protein family domain through pfam assignment [34]. The MG-RAST enzyme class assignment for reads against the SEED database was used, and the reads having CAZy EC classes were extracted [43]. In total, 102 CAZy enzyme classes (ECs) were found among sequenced reads that were assigned by MG-RAST (Additional file 4: Table S4). 18,771 reads which were classified as a CAZy target with all three methods are termed as common reads, and a total 33,503 CAZy reads were assigned by three different methods. A majority of reads shared by all three methods indicate the specificity of sequence capture from designed probes, whereas unique reads show the coverage of sequence capture over the CAZy database (Figure 6) for used agricultural soil. In this study, 331 different families from the CAZy database have been targeted for the sequence capture experiment. In the mapping result, captured sequences belong to 203 different CAZy families and 103 families are found in abundance among them (Figure 7). In total, 18,771 reads were assigned as CAZy target among all reads. A text-based search was performed over NCBI-NR BLASTX description of not-assigned reads which were found in MEGAN metabolic analysis, with the CAZy description of sequences. It was found that 59,235 reads belong to CAZy databases, but these reads don’t have a well-defined metabolic role in the KEGG database, and some reads had CAZy features yet were disqualified due to insufficient read length. This shows that the sequence capture technique has high efficiency in identifying a wide range of families in a single experiment.

Tasse *et al.* performed a clone based targeted metagenomic study which used multi-step functional approach for the investigation of carbohydrate-degrading functions in human gut metagenome. After multistep screening, 0.84 Mb of non-redundant metagenomic DNA was sequenced which correspond to 26 clones. Target gene enrichment was found to be fivefold higher than random sequencing of the human gut metagenome and 73 CAZy enzymes from 35 different families were found [45]. In our study, 0.13 Mb targeted metagenomic DNA was sequenced through designed probes from the soil metagenome of average size of 1.5 Mb - 8 Mb [46] and 203 different CAZy families have been found. Although the type and size of metagenome as well as the approach, and experimental efforts were different for both the studies, but both had the same overall goal: to investigate the functional diversity of carbohydrate degradation enzymes. Sequence capturing with probes can efficiently enhance the target enrichment several fold with less complexity and comparatively low experimental efforts. As an example, the probe design of 5,000 GI numbers from the NCBI Genbank database [47] used as input took approximately 8 hours. Probe design through direct sequence submission is the highly recommended approach for MetCap processing because it will reduce the processing time several fold which is solely depended on dataset (like how many cluster are without probes, how many sequences are in those clusters, length of sequences in cluster).

## Conclusions

The probe design tool, MetCap, takes a probe-based target enrichment approach. Compared to the alternative of primer-based enrichment, MetCap can handle a much larger set of target sequences. The probe generation was applied to a soil metagenome, and proved to be highly efficient in capturing specific target sequences. About 30% of the reads from a single metagenome matched to the targets designed by MetCap, which corresponds to an extremely high enrichment to target genes. MetCap as a web service can automate high-throughput probe generation for large datasets specifically designed for targeted metagenome sequencing projects, and ensure open access of the developed pipeline for the scientific community.

## Availability and requirements

**Project name:** MetCap

**Project home page:**http://soilecology.biol.lu.se/metcap/.

**Operating system:** web-based application (platform independent).

**Any restrictions to use by non-academics:** free for academic and non-academic users.

## Additional files

Additional file 1: Table S1. Comparative study of FGA probe generating software. Comparative study of functional gene array generating software. Additional file 2: Table S2. A comparative number of clusters with probe before and after redesigning step (IV) for Merops database. It contains the list of number of cluster with probe before and after probe regeneration step. Additional file 3: Table S3. Summary of extracted sequence from databases and generated probes. It contains the list of number of extracted sequences and their corresponding generated probes. Additional file 4: Table S4. List of CAZy enzyme classes (ECs) found among sequenced soil metagenome through MG-RAST assignment. It contains the list of CAZy enzyme classes which were found in sequenced reads.

## Abbreviation

Mb: Million bases
