# Erratum to: MetCap: A bioinformatics probe design pipeline for large-scale targeted metagenomics

**DOI:** 10.1186/s12859-015-0843-2

**Published:** 2016-01-18

**Authors:** Sandeep K. Kushwaha, Lokeshwaran Manoharan, Tejashwari Meerupati, Katarina Hedlund, Dag Ahren

**Affiliations:** Department of Biology, Lund University, Ecology Building, Lund, 223 62 Sweden; Bioinformatics Infrastructure for Life Sciences (BILS), Department of Biology, Lund University, Ecology Building, Lund, 223 62 Sweden

## Erratum

In our article [1], the numbers of probes and sequences for the CAZy dataset in the Additional file 3, table S3 (A) (Table 1 here) have been switched. The corrected table S3 (A) is presented in this erratum.

**Table 1:** (A): Family-wise summary of generated probes based on sequences downloaded from CAZy database

Family	Archaea	Bacteria	Eukaryota	Unclassified	Virus	Total probes	Total sequences
CBM	439	26910	7424	134	453	35360	**23302**
CE	311	14646	3254	66	9	18286	**13787**
GH	2754	97488	33643	1457	7382	142724	**110923**
GT	7755	106687	30802	160	683	146087	**103952**
PL	57	4663	1082	6	51	5859	**6580**
**Total**	**11316**	**250394**	**76205**	**1823**	**8578**	**348316**	**258544**

In the following three parts of our article [1] we would like to correct the numbers for probes and sequences accordingly.

### Abstract (Results), page 1

To illustrate the advantage of a targeted metagenome approach, we have generated more than 400,000 probes that match more than 300,000 publicly available sequences related to carbon degradation, and used these probes for targeted sequencing in a soil metagenome study.

### Results and discussion, page 6

In this study, 306,525 nucleotide sequences were extracted through the pipeline and used as a proof of concept. A list of group-wise collected sequences and generated probes from databases are given as Additional file 3: Table S3. In total, 406,277 unique probes were produced from these extracted nucleotide sequences in this study with the following criteria: length (50mer), GC contents (35-65), melting temperature (55-65), and 3 probes per cluster on 90 % cluster similarity.

### Results and discussion, page 8

Among the downloaded sequences (Additional file 3: Table S3), 258,544 sequences belong to the CAZy database from four major families: Glycoside Hydrolase (110,923), Glycosyl Transferases (103,952), Carbohydrate Esterases (13,787), Polysaccharide Lyases (6,580), and an associated module, Carbohydrate binding-modules (23,302) and 348,316 probes have been designed from these sequences.
