# Supplementary material for: MetCap: a bioinformatics probe design pipeline for large-scale targeted metagenomics
Source: BMC Bioinformatics. 2015 Feb 28;16(1):65. doi: 10.1186/s12859-015-0501-8 (PMC4355349; doi:10.1186/s12859-015-0501-8)
Supplement: Additional file 1: Table S1. — Comparative study of FGA probe generating software. Comparative study of functional gene array generating software. [file 12859_2015_501_MOESM1_ESM.doc]

Table S1: Comparative study of FGA producing software’s for large scale probe designing

| S.N. | Name of Software | Implemented approach in software | AT | Capacity of demonstration dataset | Probe specificity checking | Accessibility and Interface | Current  availability | RT-LD | Probe design obstacles at large scale |
| --- | --- | --- | --- | --- | --- | --- | --- | --- | --- |
| 1. | Oligonucleotide Retrieving for Molecular Applications (ORMA) | Single Base Seeker (SBS) algorithm to discriminate one sequence among a set of homologous ones | POA, FGA | 352 sequences, 16S rRNA cyanobacteria and milk-pathogens dataset | NO | Matlab script | Available on request | High | 1. Complex and time-very consuming  2. Hard to decide +ve/-ve set in large data-set  3. Tedious discrimination with highly similar and diverse sequences  4. No explorative probes |
| 2. | Hierarchical Probe Design (HPD) | MSA, Hierarchical Clustering | FGA | 421 nirS sequences and 490 pmoA sequences | Sequences of Input data-set | Downloadable, Standalone  GUI(W) | NA | NA | 1. Complex and time consuming  2. Effective for small dataset with patterns and relationships between sequences  3. loss of information is more with large data-set.  4. No explorative probes generation |
| 3. | ProDesign | Spaced seed algorithm coupled with Clustering | FGA | 51519 sequences from 11 genome of Escherichia coli for microbial community. | Sequences of Input data-set | Web-server  Downloadable, Standalone  GUI(U) | http://www.uhnresearch.ca/labs/tillier/ProDesign/ProDesign.html | Low | 1. Simple and relatively time efficient.  2. Clustering dependent coverage.  3. excellent coverage only with moderate similar sequences.  4. Lack of explorative probes |
| 4. | HiSpOD | Based on MSA and consensus sequence generation | FGA WGA | 54 enzyme sequences involved in chlorinated water degradation | EnvExBase complete CDS Database through BLAST | Web-server | http://g2im.u-clermont1.fr/hispod/index.php | Very High | 1. Simple but very time consuming.  2. No information for cluster coverage from generated probes i.e. probe generation region.  3. High computational burden due to BLAST search for individual generated probes. |
| 5. | CommOligo  (v-2.0) | Global alignments for sequence identity, free energy and continuous stretch | FGA  WGA | 842 sequences, nitrite reductase nirS and nirK genes | Sequences of Input data-set | Downloadable, Standalone GUI(W) | http://ieg.ou.edu/software.htm | Very High | 1. Very time consuming but more precise.  2. Select group-specific oligonucleotide probes for a group of very highly similar sequences.  3. Dynamic programming is slow and not better solution for large number of sequence. |
| 6. | Metabolic Design | MSA,  Back-translation | FGA | 8 genes (phnA1a, phnA2a, bphC, bphA3, ahdA1c, ahdA2c, ahdA4 and bphB) involved in the degradation of several PAHs | EnvExBase complete CDS Database through BLAST | Downloadable, Standalone GUI (W) | NA | NA | 1. Time consuming and more prone to false positive  2. High number of probe due to higher nucleotide degeneracy  3. Uncontrol explortative probe generation for highly diverse set of sequences.  4. High computational burden due BLAST Search for individual probes |

AT: Applied Type, RT-LD:Running time for large data-set (100,000), GUI: Graphical user interface, NA: Not available, FGA: Functional gene assay, MSA: Multiple Sequence Alignment, WGA: Whole genome assay, POA:Phylogenetic assays, CDS: Conserve domain sequences
