# Supplementary material for: MetCap: a bioinformatics probe design pipeline for large-scale targeted metagenomics
Source: BMC Bioinformatics. 2015 Feb 28;16(1):65. doi: 10.1186/s12859-015-0501-8 (PMC4355349; doi:10.1186/s12859-015-0501-8)
Supplement: Additional file 2: Table S2. — A comparative number of clusters with probe before and after redesigning step (IV) for Merops database. It contains the list of number of cluster with probe before and after probe regeneration step. [file 12859_2015_501_MOESM2_ESM.docx]

Table-S2: List of number of clusters with probe before and after redesigning step (IV) for Merops database.

| Merops Database | | | | |
| --- | --- | --- | --- | --- |
| Classes | Total sequences | Number of generated Cluster from sequences | Number of Cluster with probes before redesign step | Number of Cluster with probe After redesign step |
| Aspartic (A) Peptidases | 5,425 | 1,853 | 1,495 | 1,765 |
| Asparagine (N) Peptide Lyases | 537 | 314 | 305 | 314 |
| Cysteine (C) Peptidases | 19,262 | 8,682 | 1,403 | 7,779 |
| Glutamic (G) Peptidases | 119 | 74 | 73 | 74 |
| Metallo (M) Peptidases | 52,734 | 25,276 | 1,103 | 24,257 |
| Serine (S) Peptidases | 70,925 | 18,001 | 1,004 | 15,733 |
| Threonine (T) Peptidases | 4,712 | 2,220 | 1,699 | 2,169 |
| Peptidases of Unknown Catalytic Type | 3,744 | 1,479 | 1,294 | 1,461 |
