# Supplementary material for: MetCap: a bioinformatics probe design pipeline for large-scale targeted metagenomics
Source: BMC Bioinformatics. 2015 Feb 28;16(1):65. doi: 10.1186/s12859-015-0501-8 (PMC4355349; doi:10.1186/s12859-015-0501-8)
Supplement: Additional file 3: Table S3. — Summary of extracted sequence from databases and generated probes. It contains the list of number of extracted sequences and their corresponding generated probes. [file 12859_2015_501_MOESM3_ESM.doc]

**Table S3 (A): Family-wise summary of downloaded sequences from Cazy database and generated probes**

| **Family** | **Archaea** | **Bacteria** | **Eukaryota** | **Unclassified** | **Virus** | **Total**  **Sequences** | **Total**  **Probes** |
| --- | --- | --- | --- | --- | --- | --- | --- |
| CBM | 439 | 26910 | 7424 | 134 | 453 | 35360 | **23302** |
| CE | 311 | 14646 | 3254 | 66 | 9 | 18286 | **13787** |
| GH | 2754 | 97488 | 33643 | 1457 | 7382 | 142724 | **110923** |
| GT | 7755 | 106687 | 30802 | 160 | 683 | 146087 | **103952** |
| PL | 57 | 4663 | 1082 | 6 | 51 | 5859 | **6580** |
| **Total** | **11316** | **250394** | **76205** | **1823** | **8578** | **348316** | **258544** |

GH: Glycoside Hydrolases, GT: Glycosyl Transferases, PL: Polysaccharide Lyases, CE: Carbohydrate Esterases, CBM: Carbohydrate Binding Module

**Table S3 (B): Summary of extracted sequence from FOLy database and lignin degradation associated gene from NCBI and generated probes.**

| **Family** | **Total Sequence** | **Total Probes** |
| --- | --- | --- |
| FOLy + Lignin Degradation Genes | **2187** | **3166** |

Now FOLy is part of Cazy database with family name Auxiliary Activities (AAs)

**Table S3 (C): Summary of extracted sequence from Merops database and generated probes**

| **Family** | **Total Sequences** | **Total Secreted Sequences** | **Total probes from Secreted Sequence** |
| --- | --- | --- | --- |
| Aspartic (A) Peptidases | 5425 | 925 | **695** |
| Asparagine (N) Peptide Lyases | 537 | 91 | **21** |
| Cysteine (C) Peptidases | 19262 | 6726 | **6980** |
| Glutamic (G) Peptidases | 119 | 66 | **113** |
| Metallo (M) Peptidases | 52734 | 14170 | **16401** |
| Serine (S) Peptidases | 70925 | 22345 | **29112** |
| Threonine (T) Peptidases | 4712 | 1375 | **1514** |
| Peptidases of Unknown Catalytic Type | 3744 | 96 | **71** |
| **Total** | **157458** | **45794** | **54907** |
